# Suicide and Suicidal Ideation Among Bhutanese Refugees — United States, 2009–2012

**Published:** 2013-07-05

**Authors:** Jennifer Cochran, Paul L. Geltman, Heidi Ellis, Cheryl Brown, Stephanie Anderton, Jessica Montour, Monica Vargas, Kenneth Komatsu, Carrie Senseman, Barbara Lopes Cardozo, Teresa I. Sivilli, Curtis Blanton, Sharmila Shetty, Eboni Taylor, Emily Lankau, Trong Ao

**Affiliations:** Refugee Health Technical Assistance Center, Massachusetts Dept of Public Health; Boston Children’s Hospital and Harvard Medical School; New York State Dept of Health; Texas Dept of State Health Svcs; Georgia Dept of Public Health; Arizona Dept of Health Svcs; Div of Global Health Protection, Center for Global Health; Div of Global Migration and Quarantine, National Center for Emerging and Zoonotic Infectious Diseases; EIS officers, CDC

During the period February 2009–February 2012, the Office of Refugee Resettlement of the U.S. Department of Health and Human Services reported 16 suicides among the approximately 57,000 Bhutanese refugees who had resettled in the United States since 2008. In 2012, the office requested assistance from CDC and the Massachusetts Department of Public Health’s Refugee Health Technical Assistance Center to identify risk factors that might be associated with suicidal ideation among Bhutanese refugees. In collaboration with the Massachusetts refugee health center, CDC conducted a survey of randomly selected Bhutanese refugees in four U.S. states with large populations of resettled refugees. The results indicated significant associations between ever having expressed suicidal ideation and current self-reported symptoms of mental health disorder (e.g., anxiety, depression, or posttraumatic stress disorder) and postmigration difficulties (e.g., family conflict or inability to find work). The findings highlight the need for development of culturally appropriate community-based interventions for suicide prevention and standard procedures for monitoring and reporting suicides and suicide attempts in the Bhutanese refugee population.

## Suicide Rate Among Bhutanese Refugees

Based on the 16 reported suicides (four in 2009, six in 2010, five in 2011, and one as of February 2012), the annual suicide rate among Bhutanese refugees resettled in the United States was calculated by investigators as 21.5 per 100,000; the age-adjusted suicide rate using the U.S. 2000 population as the standard was 24.4 per 100,000. Both estimates were higher than the estimated annual global suicide rate for all persons of 16.0 per 100,000 (1) and the annual suicide rate for U.S. residents of 12.4 per 100,000 (2), but were similar to the prearrival suicide rate in Bhutanese refugee camps in Nepal of 20.7 per 100,000 (3).

## Assessment of Suicidal Ideation

After stratifying by state, a sample of 579 Bhutanese refugees aged ≥18 years living in Arizona, Georgia, New York, and Texas was randomly selected. Trained bilingual and bicultural interviewers contacted the potential participants, obtained written informed consent, and administered the survey in the respondent’s home using the respondent’s preferred language (English or Nepali). The survey asked about demographics, mental health history, difficulties after arrival in the United States, perceived level of social support (4), current symptoms of posttraumatic stress disorder (PTSD) and traumatic experiences (using the Harvard Trauma Questionnaire [5]), and symptoms of anxiety, depression and psychological distress (using the Hopkins Symptom Checklist [6]). Participants also were provided information on local mental health services and were encouraged to access these services if needed.

Participants were asked if they had ever expressed suicidal ideation (i.e., ever thought seriously about committing suicide in their lifetimes). Interviewers were trained to implement a distressed respondent protocol if a participant expressed suicidal thoughts during the interview. Data were summarized and tested for statistically significant differences between men and women using the chi-square test for categorical variables and t-test for continuous variables. Adjusted bivariate associations (by age, sex, and state of residence) between suicidal ideation and other variables were estimated with adjusted odds ratios, associated Wald chi-square tests, and 95% confidence intervals, using conditional logistic regression.

The response rate for the survey was 73% (423 of 579). Of the 423 participants, 221 (52%) were men. Most (72%) were married, Hindu (72%), and had a regular income (65%). Median age was 34 years (range: 18–83 years), and median time in the United States was 1.8 years (range: 0.2–5.0 years). A total of 148 (35%) participants had no education, 56 (13%) had no more than a primary education, 163 (38%) had attended a secondary school, and 54 (13%) had a university or graduate degrees. Fifteen (4%) participants reported ever having been diagnosed with a mental health disorder. Seventy-nine (19%) had current anxiety symptoms (15% of men, compared with 23% of women, p=0.04); 82 (20%) had current depressive symptoms (16% of men, compared with 26% of women, p=0.01), and 69 (17%) had current psychological distress symptoms (13% of men, compared with 23% of women, p=0.01). Using a scoring algorithm created by the Harvard Refugee Trauma Group based on the PTSD symptom criteria from the *Diagnostic and Statistical Manual of Mental Disorders, Fourth Edition, Text Revision* (DSM-IV-TR), the prevalence of PTSD symptoms was estimated at 5% (3% of men, compared with 6% of women, p=0.17).

A total of 153 (36%) participants reported experiencing four to seven presettlement traumatic events or significant stressors, and 145 (34%) reported experiencing eight or more traumatic events or stressors. The most common traumatic event was lack of nationality or citizenship (90%), followed by having to flee suddenly (54%), and lack of freedom of movement (52%). Commonly reported postarrival difficulties were language barriers (62%), lack of choice (46%), and worries about family back home (39%).

Of the 423 participants, 131 (30%) had personally known someone who had taken their own life; of the 131, a total of 24 (18%) had been emotionally close to the suicide decedents. Thirteen (3%) of the 423 participants reported that they had ever expressed suicidal ideation. Of these, nine had thought about it in the past 12 months, three had once made a plan, and one had attempted suicide. One participant expressed suicidal thought during the interview, and the appropriate distressed respondent protocol was implemented to provide care for this participant.

Respondents who were not providers for their family were more likely (adjusted odds ratio [AOR] = 6.6) to have ever expressed suicidal ideation than family providers (i.e., persons expected to be financially responsible for the family, regardless of current employment status) (Table). Self-reported symptoms of anxiety (AOR = 38.1), distress (AOR = 15.0), and depression (AOR = 11.2) were strongly associated with ever expressing suicidal ideation, compared with those without those symptoms. Those categorized as reporting symptoms of PTSD were more likely to report suicidal ideation than those without PTSD (AOR = 9.3). Among postarrival difficulties faced by refugees, increased family conflict (AOR = 22.6) and being unable to find work (AOR = 11.1) were the difficulties most strongly associated with suicidal ideation (Table).

### Editorial Note

Since the early 1990s, approximately 100,000 Bhutanese of Nepali origin (*Lhotshampas*) have been living in refugee camps in Nepal because of cultural and religious persecution in Bhutan. Third-country resettlement began in 2008, and to date, approximately 57,000 Bhutanese refugees have been resettled in the United States. Since the study described in this report was concluded, four additional suicides have been reported among Bhutanese refugees in the United States (U.S. Department of Health and Human Services, Office for Refugee Resettlement, unpublished data, 2013). Currently, reporting of suicides and suicide attempts among Bhutanese refugees is through informal channels of communication, including the community, resettlement agencies, state refugee health coordinators, and the Office for Refugee Resettlement. A timely reporting system that accurately obtains information about suicide and suicide attempts in these communities is needed to enable appropriate supportive care for the families and community affected.

Although prearrival and postarrival suicide rates among Bhutanese refugees appear similar, different psychological stressors occur at each stage of the resettlement process. This study identified postarrival difficulties (e.g., being unable to find work and increased family conflict) and symptoms of anxiety, depression, and psychological distress as factors significantly associated with having ever expressed suicidal ideation. Both continuing those interventions already implemented to address the prearrival risk factors in the Nepal refugee camps (e.g., maintaining peer-support groups and providing informal counseling sessions with community psychosocial workers) and addressing these postarrival difficulties and symptoms are important to a comprehensive suicide prevention strategy.

Although only 4% of respondents reported being previously diagnosed with a mental health disorder, this investigation identified much higher prevalences of current anxiety, depression, and distress symptoms (19%, 21%, and 17%, respectively), with significantly higher proportions among women. This might suggest high levels of undiagnosed mental health disorders in these communities. For comparison, the prevalence of current self-reported depression among adults in the United States was approximately 8% in the National Health and Nutrition Examination Survey during 2007–2010 (7), and the prevalence of self-reported depression was 15.1% in a population-based study in Chennai, India (8).

The findings in this report are subject to at least three limitations. First, suicide and mental health are inherently sensitive topics; therefore, reported mental health disorders and suicidal ideation and suicide attempts might have been underreported. Because no structured clinical interviews were conducted, the extent to which self-reported symptoms of PTSD, psychological distress, depression, and anxiety might be matched by clinical diagnoses is uncertain. In addition, cultural or religious perspectives on suicide were not explored, and an understanding of these might have provided additional context for interpretation of the accuracy of the data. Second, the cross-sectional study design did not allow inference of causal relationships between the risk factors and expression of suicidal ideation. Finally, the 73% response rate might have resulted in bias. However, when the characteristics of the participants were compared with those for the U.S. population of Bhutanese refugees, no marked differences were observed. Nonetheless, these results, drawn from data in four states, are not generalizable to other Bhutanese populations inside and outside of the United States or to other refugee populations.

What is already known on this topic?

Mental health and suicide among Bhutanese in refugee camps in Nepal are growing public health concerns.

What is added by this report?

Sixteen suicides among U.S.-resettled Bhutanese refugees were reported to the Office of Refugee Resettlement during February 2009–February 2012. The age-adjusted incidence of suicide among Bhutanese refugees resettled in the Untied States was 24.4 per 100,000. Expression of suicidal ideation was reported by 3% of respondents. Suicidal ideation was significantly associated with having symptoms of mental illness and postarrival difficulties such as family conflict and being unable to find work.

What are the implications for public health practice*?*

These findings suggest that Bhutanese refugees who have resettled in the United States could have a high percentage of undiagnosed mental illness. Prioritizing mental health services might be important to the successful resettlement of Bhutanese refugees in the United States. Current programs that address postarrival challenges such as job training and language training should consider adding social support and mental health components. Refugee communities and service providers might benefit from additional suicide awareness training to identify those at greatest risk and greatest need for early intervention.

Based on the findings of this investigation, the following strategies might be important in creating a comprehensive suicide prevention plan in these communities: 1) immediately follow up with the recent suicides to connect affected families and communities with supportive services; 2) integrate cultural brokers (i.e., Bhutanese refugee community leaders who act as a liaison between community members and service providers) into existing mental health services to promote language and cultural access for refugees; 3) engage the suicide prevention coordinator in each state to facilitate linkages between refugee communities/resettlement networks and suicide prevention services; and 4) follow the CDC Recommendations for a Community Plan for the Prevention and Containment of Suicide Clusters and Recommendations for Reporting on Suicide (9) when there is a cluster of suicides in a community.

In addition to predaparture suicide prevention strategies already implemented by the International Organization for Migration in Bhutanese refugee camps (3), this report highlights the need for further suicide prevention activities in the United States that might include providing 1) training for suicide prevention gatekeepers (i.e., anyone who comes into regular contact with distressed persons or families); 2) other nonclinical community support interventions in Bhutanese community activities, such as religious singing groups and sports teams; and 3) standardized and coordinated reporting of information on confirmed suicides or suicide attempts.

## Figures and Tables

**TABLE:** Adjusted odds ratios (AORs)* for ever expressing suicidal ideation, among Bhutanese refugees (N = 423) resettled in the United States, by selected characteristics, 2012

Characteristic	Suicidal ideation		No suicidal ideation		AOR	(95% CI)
	
	No. (n = 13)	(%)	No. (n = 404)	(%)		
Nonprovider for family	11	(84.6)	204	(50.5)	6.6	(1.4–31.9)
Anxiety†	11	(84.6)	67	(16.6)	38.1	(7.9–185.1)
Depression†	8	(66.7)	74	(19.4)	11.2	(2.9–42.1)
Distress†	8	(66.7)	60	(15.8)	15.0	(3.9–57.1)
Posttraumatic stress disorder§	3	(23.1)	16	(3.9)	9.3	(2.1–41.0)
Experienced burning down of house or shelter	7	(53.9)	105	(26.0)	3.4	(1.1–10.3)
**Postmigration experience**						
Increased family conflict	4	(30.8)	8	(1.9)	22.6	(5.5–92.6)
Being unable to find work	11	(84.6)	145	(35.9)	11.1	(2.4–51.5)
Poor access to counseling services	8	(61.5)	75	(18.6)	7.9	(2.5–25.4)
Lack of community structures for family dispute	3	(23.1)	30	(7.4)	4.8	(1.2–19.8)
Lack of choice over future	10	(76.9)	185	(45.8)	4.7	(1.2–17.8)
Little help from government	8	(61.5)	125	(30.9)	3.6	(1.2–11.4)
**Coping mechanism**						
Wished people would just leave you alone	5	(38.5)	23	(5.7)	14.5	(3.9–52.8)
Thought about what needed to be done	11	(84.6)	205	(50.7)	7.0	(1.5–33.1)
Talked with community leaders or elders	4	(30.8)	49	(12.1)	3.4	(1.0–11.7)

**Abbreviation:** CI = confidence interval.

* Adjusted for state of residence, age, and sex.

† Based on the Hopkins Symptom Checklist.

§ Defined as at least one of four reexperiencing symptoms in addition to at least three of seven avoidance and numbing symptoms, and at least two of five arousal symptoms.

## References

[1] World Health Organization (2013). Suicide prevention (SUPRE).

[2] CDC (2013). Web-based Injury Statistics Query and Reporting System (WISQARS).

[3] Schinina G, Sharma S, Gorbacheva O, Mishra AK, International Organization for Migration Who am I? Assessment of psychosocial needs and suicide risk factors among Bhutanese refugees in Nepal and after third country resettlement, 2011.

[4] Cutrona CE (1986). Objective determinants of perceived social support. J Pers Soc Psychol.

[5] Mollica RF, Caspi-Yavin Y, Bollini P, Truong T, Tor S, Lavelle J (1992). The Harvard Trauma Questionnaire: validating a cross-cultural instrument for measuring torture, trauma, and posttraumatic stress disorder in Indochinese refugees. J Nerv Ment Dis.

[6] Shrestha NM, Sharma B, Van Ommeren M (1998). Impact of torture on refugees displaced within the developing world: symptomatology among Bhutanese refugees in Nepal. JAMA.

[7] National Health and Nutrition Examination Survey, 2007–2010.

[8] Poongothai S, Pradeepa R, Ganesan A, Mohan V (2009). Prevalence of depression in a large urban South Indian population—the Chennai Urban Rural Epidemiology Study (CURES-70). PLoS One.

[9] CDC (1988). CDC recommendations for a community plan for the prevention and containment of suicide clusters. MMWR.

